# Hand, Foot, and Mouth Disease Challenges and Its Antiviral Therapeutics

**DOI:** 10.3390/vaccines11030571

**Published:** 2023-03-01

**Authors:** Zijie Li, Wangquan Ji, Shuaiyin Chen, Guangcai Duan, Yuefei Jin

**Affiliations:** 1 Department of Epidemiology, College of Public Health, Zhengzhou University, Zhengzhou 450001, China; 2 Henan Key Laboratory of Molecular Medicine, Zhengzhou University, Zhengzhou 450001, China

## 1. Introduction

**Figure 1:**
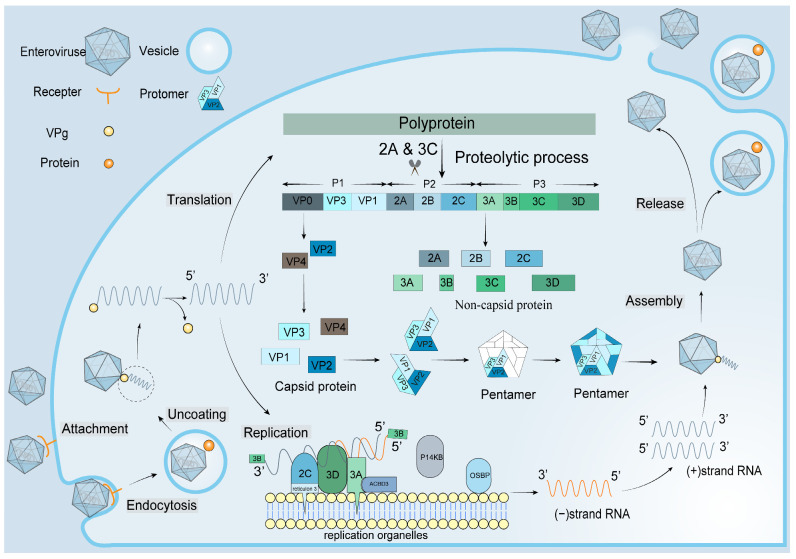
Schematic overview of EV life cycle.

Hand, Foot, and Mouth Disease (HFMD) is an infectious disease caused by enteroviruses (EVs) and is extremely contagious and prevalent among infants and children under 5 years old [1]. Historically, outbreaks of HFMD have been caused primarily by enterovirus A71 (EVA71) and coxsackievirus A16 (CVA16). Recently, however, other EV serotypes (e.g., CVA10, CVA6, CVA2, CVA8) have been associated with an increasing number of HFMD outbreaks and sporadic cases globally. Similar to other viruses, the life cycle of EVs includes attachment, endocytosis, uncoating, translation, replication, assembly, and release (Figure 1) [2].

HFMD has caused several large outbreaks worldwide and has created a huge disease burden in the world, especially in the Asia-Pacific region [1,3,4]. The main manifestations of HFMD are fever, vesicular rashes on hand, feet and buttocks, and ulcers in the oral mucosa. Although usually mild and self-limiting, HFMD can occasionally cause severe complications associated with the central nervous system or fatal respiratory disease [5]. However, there are no effective antiviral drugs available for HFMD, and supportive care as well as symptomatic treatment are currently the main treatment strategies for critical cases [6]. Although three kinds of monovalent inactivated EVA71 vaccines have been licensed by China FDA, these vaccines cannot provide broad-spectrum protection for other EV serotypes [2,7]. Polyvalent vaccines and other types of vaccines (e.g., virus-like particles vaccines, viral protein subunit vaccines, recombinant VP1 and P1 vaccines) are currently under development [7,8]. Facing the severe situation of HFMD epidemic, it is urgent to develop antiviral drugs for HFMD. This Editorial summarizes the current developments in antiviral agents for HFMD and highlights the viable research directions of antiviral agents.

## 2. Recent Development in the Design of Antiviral Agents

To date, proteins and structures that play an important role in the life cycle of EVs have been widely studied as drug targets. An increasing number of compounds acting on these drug targets are being identified as potential antivirals for HFMD. The following is a summary of recent researches in this area.

### 2.1. Antiviral Agents Targeting the Five-Fold Axis

Virus-host receptor interaction is the first essential event during virus infection [9]. At least six host cellular receptors can be involved in this process, such as scavenger receptor class B member 2 (SCARB2), P-selectin glycoprotein ligand-1 (PSGL-1), nucleolin and cell surface heparan sulfate (HS). Conserved and positively charged amino acid clusters (such as Arg166 and Lys242) are symmetrically arranged near the five-fold axis and serve as binding sites for cellular receptors [10]. It has been discovered that these amino acids interact with drugs to effectively prevent viral attachment to the relevant receptors [11].

A group of negatively charged glycosaminoglycans, including heparin, polysulfated dextran sulfate and suramin, can prevent EVA71 infection. They interact with the positively charged amino acid cluster of EVA71 and affect the attachment of EVA71 to HS [12]. A peptide derived from EVA71 VP1, L-SP40 peptide, was found to exhibit significant antiviral activity against EVA71 by blocking viral attachment to nucleolin [13]. Moreover, it demonstrated 80% protection of neonatal mice against lethal challenge by EVA71 [13]. Sulforaphane derivative NF449, rosmarinic acid, and a series of tryptophan dendritic macromolecules specifically block EV interaction with PSGL-1 as well as HS and inhibit EV attachment to target cells [14,15,16]. Transmembrane protein 106A (TMEM106A) is a protein produced by interferon (IFN) stimulation. TMEM106A is required for IFN-mediated viral suppression and interferes with the binding of EVA71 to SCARB2 [17]. A number of sulphonated azo dyes, widely used as food additives, have been identified as having potent antiviral activity against EVs, Brilliant black BN (E151) being one of them. E151 interacts with the apex of the five-fold axis to prevent viral entry and is regulated by amino acids at VP1-98, 145, and 246 [18]. An extract of Ilex kaushue, 3,4-dicaffeinated quinic acid (3,4-DCQA), targets the five-fold axis to form a stable structure with the VP-98 and 246 residues through noncovalent and van der Waals interactions, specifically inhibiting the attachment of EVA71 to the host receptor HS [19].

### 2.2. Antiviral Agents Targeting the Hydrophobic Pocket

The circular depression around the five-fold axis is called canyon, which has been shown to bind to cellular receptors. When the cell receptor binds to the canyon, the receptor presses against the bottom of the canyon, which corresponds to the top of the pocket, leading the hydrophobic pocket to release pocket factor (PF). PF plays an important role in regulating particle stability, and the release of PF leads to viral uncoating and genome release, which is the switch initiating EV infection [20,21]. Similarly, when antiviral bind to the pocket, it also expands the bottom of the canyon and affects receptor binding [22]. Antivirals competing for the same binding site usually have a higher affinity than PF [23]. Therefore, antivirals can bind to the hydrophobic pocket and inhibit viral uncoating, whether the pocket contains PF or not [21]. 

The WIN series from Sterling-Winthrop is a prominent series of capsid-binding compounds. WIN compounds and their derivatives, including WIN54954, WIN51711, BPR0Z-194, pleconaril, vapendavir, and pirodavir, have anti-EVs capabilities [21,24,25,26,27]. Among these, the broad spectrum EVs inhibitor pleconaril is the most potent antiviral, with a 50% inhibition Concentration (IC_50_) of 0.014 μg/mL against coxsackievirus B3 (CVB3) infection [26]. However, pleconaril is ineffective against EVA71 [28]. Two new compounds, NLD and ALD, designed based on the crystallographic analysis of the structure of 3-(-4-pyridyl)-2-imidazolidinone derivatives GPP3, and show good activity against both EVA71 and CVA16. NLD and ALD inhibited EVA71 replication with IC_50_ of 0.025 and 8.54 nmol/L, making them the most potent EVA71 inhibitors reported so far [29]. An imidazolidinone derivative, PR66, protected EVA71-induced neurological symptoms in a mouse model [30]. 

### 2.3. Antiviral Agents Targeting Non-Structural Proteins of EVs

The 2A protein (2Apro) catalyzes the peptide bond cleavage between VP1 and P2. Through 2Apro, EVA71 cleaves eukaryotic translation initiation factor 4G (eIF4G) and hijacks the cellular translation machinery to produce viral proteins [31]. Meanwhile, it was found that blocking the RAS/RAF/MEK/ERK (MAPK) pathway would inhibit the hydrolytic activity of 2Apro [32]. Therefore, direct targeting of 2Apro and inhibiting the MAPK pathway are how these inhibitors exert antiviral effects. 

A 6-amino-acid hit peptide LVLQTM, and a novel furoquinoline alkaloid CW-33, inhibit EVA71 activity by directly targeting 2Apro [33,34]. Sorafenib, a clinically approved anti-cancer multi-targeted kinase inhibitor, blocks the MAPK pathway and significantly inhibits the hydrolytic activity of EVA71 2Apro [35]. Researchers designed and synthesized a series of substituted 3-benzylcoumarins, two of which compounds 13 and 14, were more effective in inhibiting MAPK pathway activity and had stronger antiviral effects compared to sorafenib [31].

The 2Cpro is one of the highly conserved viral proteins in all picornaviruses and functions as ATPase, 3’-5’ ATPase-dependent RNA helicase and ATPase-dependent RNA chaperone. In addition, 2Cpro is believed to involve in the formation of replicative organelles (ROs) of the virus and innate immune evasion [36].

Mutations in the coding sequence of the CVB3 2Cpro conferred resistance to dibucaine, pirlindole, fluoxetine and zuclopenthixol, suggesting that 2Cpro is the target for this set of compounds [37]. A peptide named 2CL was designed based on the structure of EVA71 2Cpro, which effectively impaired the oligomerization of EVA71 2Cpro and inhibited the RNA helicase activities of 2Cpro encoded by EVA71 and CVA16 [38]. An aryl-substituted amide, R523062, inhibits multiple strains of EVA71. Thermal displacement binding assays showed that R523062 binds to 2Cpro [39]. Moreover, some 1H-pyrazolo[3,4-b]pyridine-4-carboxamide molecules and dibucaine derivatives are also considered to be effective 2Cpro inhibitors [40,41].

3Apro is a membrane-bound protein that plays an important role in membrane rearrangement by recruiting essential host factors to ROs [36]. Enviroxime is a broad-spectrum anti-EVs drug targeting 3Apro. However, the clinical development was halted due to the lack of therapeutic benefits and gastrointestinal side effects [42]. Subsequently, a series of enviroxime analogs were synthesized and used for anti-EVs. The best-known of these compounds are GW5074, TTP-8307, and T-00127-HEV2 (THEV) [43]. Some FDA-approved drugs (such as itraconazole and posaconazole) have also been found to be effective 3Apro inhibitors [44,45].

3Cpro is responsible for the proteolytic processing of the Gln-Gly peptide bond be- tween P2–P3, resulting in the production of nonstructural and structural proteins [46]. 3Cpro was found to possess RNA-binding activity. Mutations in 3Cpro influenced RNA-binding activity and proteolytic activity [36]. In addition, 3Cpro can regulate cell pyroptosis and apoptosis through different pathways [36].

Rupintrivir (AG7088) represents a typical peptidyl mimic, it has an α,β-unsaturated ester group as the functional group [47], which functions as a Michael acceptor for nucleophilic Cys residues in the catalytic center of 3Cpro and affects 3Cpro activity [48]. Molecular docking experiments confirmed that Quercetin and two cyanohydrin derivatives FOPMC, and FIOMC, bind non-covalently to 3Cpro and coordinate with surrounding amino acid residues through hydrophobic interactions [49,50]. NK-1.8k and NK-1.9k are two peptide aldehydes identified as potent and selective inhibitors targeting EV 3Cpro [47,51]. SLQ-4 and SLQ-5 are EVA71 3Cpro inhibitors with two strong electron-withdrawing groups (EWGs), the two EWGs confer excellent potency to the Michael receptor, allowing the drug to inhibit viral activity more effectively [46].

3Dpro, as an RNA-dependent RNA polymerase (RdRp), is responsible for RNA replication. EVA71 mediates cell cycle arrest in S-phase through 3Dpro. In addition, 3Dpro is involved in VPg uridylation and has been shown to play an important role in the activation of inflammatory responses as well as evasion of innate immunity [36]. Both NITD008 and FNC are nucleoside analogs that can effectively inhibit EVA71 by targeting and inhibiting 3Dpro activity [52,53]. However, as nucleoside analogs, these drugs may have many side effects. DTriP-22 was identified as a non-nucleoside analog that targeted 3Dpro. It inhibits the poly (U) elongation activity of EVA71 polymerase [54], but not the VPg uridylation activity. Differently, another 3Dpro inhibitor, BPR-3P0128, inhibited both of these activities [55].

### 2.4. Antiviral Agents Targeting Internal Ribosome Entry Site (IRES)

The 5′ untranslated region (5′UTR) is the most phylogenetically conserved portion of the EVs genome, it folds into a structured RNA element composed of six stem-loops. The first stem-loop (SLI) directs RNA strand synthesis, and stem-loop II-VI constitutes IRES, which promotes cap-independent translation by internally recruiting the 40S ribosomal subunits [56]. In contrast to host cap-dependent cellular translation, the genome of EVs lacks a 50 cap, so EVs initiate viral RNA translation through IRES. Highly structured IRESs have also been used to identify virus inhibitors [57].

A flavonoid, kaempferol, was found to affect IRES function and EVA71 replication by altering the composition of IRES trans-acting factors (ITAFs) [58]. It suggested that the mechanism of IRES inhibitors was generated by affecting the interaction of viral RNA with ITAFs. Apigenin interacts with ITAF heterogeneous nuclear ribonucleoproteins (hnRNPs) and interferes with viral RNA editing activity [59]. Multiple binding of Idarubicin to EVA71 5′UTR RNA impairs binding between EVA71 IRES RNA and the ITAF hnRNPA1, resulting in a significant reduction in IRES activity [60]. The cellular proteins, AU-rich element factor 1 (AUF1), bind to the bulge of SLII to repress IRES-dependent translation [61]. Pull-down experiments in cell culture support that the EVs inhibitor DMA-135 induces a conformational change in virus RNA structure that stabilizes a ternary complex with the AUF1 protein, thus repressing translation [62].

## 3. Prospects and Summary

The functions of proteins and structures that act as drug targets have been extensively studied. With the advent of novel drug screening methods, as well as drug synthesis methods, more and more drugs have been recognized as broad-spectrum inhibitors of EVs. Unfortunately, most drugs will fail at some stages of clinical development due to off-target effects and/or lack of antiviral efficacy [63]. Childhood infections with milder symptoms are the main group of HFMD [5]. Thus, more attention should be paid to the side effects and toxicity of the drugs. Screening drugs that have passed clinical trials is a good approach, but further safety assessment trials are still needed. Many plants have antiviral and anti-inflammatory properties, especially those with medicinal value, such as herbs. Natural compounds extracted from these plants are often very effective against HFMD [64]. Many previous researches had been conducted on antiviral drugs for EVA71 and CVA16. However, as the pathogenic spectrum of HFMD has changed in recent years, the development of broad-spectrum antiviral drugs is urgently required, especially for important HFMD pathogens such as CVA6, CVA10, CVB3, etc. At the same time, for existing drugs, researchers can improve the pharmacokinetic characteristics and efficacy of the drug by changing parts of the drug’s structure, based on studies about the drug’s structure. Resistant mutants have been selected for almost all direct-targeting antivirals in cell culture, suggesting that most drugs exhibit a low resistance barrier. Selecting drugs that do not make the virus resistant is also an issue that needs attention in future research. More and more researchers are no longer targeting viral proteins as drug targets but instead targeting host factors, which solves the problem of drug resistance effectively.

Collectively, HFMD is a major threat to global public health, especially in the Asia-Pacific region. Although much progress has been made in the research of effective antiviral agents for HFMD, there is still a long way to go before clinical trials can be adopted and truly benefit patients.
